# Effects of intensive blood‐pressure treatment on myocardial work in elderly hypertensive patients: A subcenter study of the STEP randomized controlled trial

**DOI:** 10.1002/clc.24172

**Published:** 2023-10-11

**Authors:** Xiaoxuan Feng, Mengqi Yan, Linghui Tang, Dan Zhou, Shiping Wu, Jun Cai, Yingqing Feng

**Affiliations:** ^1^ lnstitute of Hypertension, Guangdong Provincial People's Hospital (Guangdong Academy of Medical Sciences) Southern Medical University Guangzhou China; ^2^ Department of Cardiology, Guangdong Provincial People's Hospital's Nanhai Hospital the Second People's Hospital of Nanhai District Foshan City Foshan China; ^3^ Department of Internal Medicine Shenzhen People's Hospital (The Second Clinical Medical College, Jinan University; the First Affiliated Hospital Southern University of Science and Technology) Shenzhen China; ^4^ Hypertension Center, Fuwai Hospital, State Key Laboratory of Cardiovascular Disease of China, National Center for Cardiovascular Diseases of China Chinese Academy of Medical Sciences and Peking Union Medical College Beijing China

**Keywords:** elderly hypertension, intensive blood pressure treatment, left ventricular, myocardial work

## Abstract

**Background:**

The benefits and safety of intensive blood pressure treatment in elderly hypertensive patients have been proved in the STEP trial. However, relevant mechanisms for intensive treatment are lacking.

**Hypothesis:**

We aimed to explore whether intensive blood pressure treatment is associated with left ventricular systolic function changes as evaluated by myocardial work (MW) parameters in elderly hypertensive patients compared to the standard.

**Methods:**

Patients were randomized to the intensive group (*n* = 66, median age 66 years, 42.4% male) with a systolic blood pressure (SBP) goal of 110 to <130 mmHg or the standard treatment group (*n* = 50, median age 63.5 years, 30% male) with an SBP goal of 130–<150 mmHg in this subcenter study of the STEP trial. There was no pre‐randomization echocardiographic collected. Echocardiographic exam was produced at 1‐year (phase 1) and 3‐year (phase 2) post‐randomization.

**Results:**

In phase 1, SBP was already significantly lower in the intensive treatment group than in the standard treatment group (126.5 vs. 132.1 mmHg, *p* < .05). During a median follow‐up of 40 months, in phase 2, the intensive group still had a lower SBP than the standard treatment group (125.0 vs. 135.3 mmHg, *p* < .05). Both global work index (GWI) and global constructive work (GCW) decreased significantly in phase in the intensive treatment group but not in the standard group (*p* < .05). Global wasted work (GWW) increased and global work efficiency (GWE) declined in both groups from phase 1 to phase 2 while no significant difference between the treatment effects. Similarly, left ventricular ejection function (LVEF) and global longitudinal strain (GLS) decreased in the two groups. The multivariate linear regression analysis showed the intensive treatment appeared to be an independent predictor of the ΔGWI (*β* = −110.92; 95% CI, −197.78 to −30.07, *p* = .008) and ΔGCW (*β* = −135.11; 95% CI, −220.33 to −49.88, *p* = .002).

**Conclusions:**

In elderly hypertensive patients, lower SBP was associated with decreased GWI and GCW and intensive BP treatment did not improve global MW efficiency.

## Graphical abstract

We investigated MW parameters and intensive antihypertensive treatment. We found that intensive antihypertensive treatment significantly reduced GWI and GCW from phase 1 to phase 2. Both treatment groups’ GWW increased and GWE decreased from phase 1 to phase 2. Intensive antihypertensive treatment did not reverse myocardial work in elderly hypertensives.

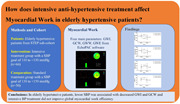

## 1 INTRODUCTION

Hypertension sheds light on hypertensive heart disease (HHD) with ventricular hypertrophy processes, systolic and diastolic dysfunction, and a wider range of cardiac and vascular adaptive responses for performance.^1^ Due to the age‐related changes in the vasculature and heart, elderly hypertension is associated with increased risks of cardiovascular events and cardiac dysfunction.^2^, ^3^ Therefore, monitoring the alternations in myocardial function attaches great importance in elderly hypertensive patients. It has been proved that hypertensive patients have impaired left ventricular (LV) systolic function early as determined by strain, which is associated to mortality, heart failure, myocardial infarction, and stroke.^4^

Left ventricular pressure‐strain loop (LV‐PSL) non‐invasively evaluates LV systolic function, which was first proposed by Russell et al.^5^ From the perspective of cardiac energetics, the LV‐PSL reflects the myocardial metabolism and oxygen consumption with consistency in F‐fluorodeoxyglucose positron emission tomography (PET) description.^5^ LV‐PSL combining global longitudinal strain (GLS) by two‐dimensional speckle tracking technology (2D‐STE) and estimated LV pressure based on blood pressure (BP) is the measurement of myocardial work (MW), which outperforms both GLS and left ventricular ejection fraction (LVEF) in addressing the afterload‐dependence deficit.^6^ Multivariable analysis showed systolic blood pressure (SBP) but not diastolic blood pressure (DBP) was an independent factor for MW parameters in EACVI NORRE study.^7^ MW was also considered to perform more sensitively than layer‐specific GLS in detecting subclinical LV systolic dysfunction among the essential hypertensive patients without LV hypertrophy.^8^

According to the results from the Systolic Blood Pressure Intervention Trial (SPRINT) and previously published research in the Strategy of Blood Pressure Intervention in the Elderly Hypertensive Patients (STEP) trial, intensive BP control reduced cardiovascular events and had adequate safety in elderly hypertensives.^9^, ^10^ However, little information is known about how these two treatments affect subtle changes in left ventricular systolic function as measured by MW parameters, which could offer insights into cardiac event pathogenesis. Therefore, the present STEP subcenter study aims to (a) examine association of MW parameters and intensive BP treatment; and (b) investigate whether MW parameters are more sensitive to changes during intensive BP treatment than to traditional parameters.

## 2 METHODS AND MATERIALS

### 2.1 Study cohort

We prospectively recruited elderly hypertensive patients from the Guangdong clinical center for the STEP trial between May 2017 and January 2018. Previous research detailed the trial protocol and main findings.^9^ Briefly, STEP was a randomized clinical trial conducted at 42 clinical centers throughout China of BP‐lowering treatment in 8511 participants aged 60–80 with abnormal SBP (140–190 mmHg) during three screening visits or taking antihypertensive medication. The patients were subsequently randomized 1:1 to either the intensive treatment group (with an SBP goal of 110–<130 mmHg) or the standard treatment group (with an SBP goal of 130–<150 mmHg).

When BP control was achieved, patients volunteered for an echocardiogram at the 1‐year (phase 1) and 3‐year (phase 2) follow‐up after randomization. Severe valvular disease, cardiomyopathy, congenital heart disease, and arrhythmia were excluded. The (Supporting Information: Table S1) STEP Supplementary Appendix lists all inclusion and exclusion criteria.^9^ In total, 116 hypertensive patients (66 intensive, 50 standard) received complete echocardiographic data collection and images at both phase 1 and 2, and analyses were based on these individuals (Supporting Information: Figure S1). The current study at Guangdong Provincial People's Hospital has been approved by the Clinical Research Ethics Committee and all patients provided written informed consent.

### 2.2 Anthropometry and biochemistry

Physicians gathered baseline demographic (age and sex) and anthropometric information (height and weight) during face‐to‐face recruitment visits. Standardized questionnaires were used to collect data on smoking status, duration of hypertension, medical history, and medication history. Weight in kilograms divided by height in meters squared yielded body mass index (BMI). Body surface area (BSA) was calculated by the Stevenson formula. After at least 5 minutes of rest, clinic BP readings were averaged from three consecutive measurements in the seated position taken 1 minute apart at baseline and two phases before echocardiographic evaluation. The ranges for SBP are derived from the baseline distribution in thirds. Additionally, a baseline biochemical test comprised fasting serum glucose, lipid profile consisting of total cholesterol (TC), triglycerides (TG), high‐density lipoprotein cholesterol (HDL‐C), low‐density lipoprotein cholesterol (LDL‐C), along with kidney‐related marker creatinine. The MDRD algorithm for Chinese was used to determine eGFR. Renal dysfunction was defined as eGFR <60 mL/min per 1.73 m^2^.

### 2.3 Conventional echocardiographic parameters

Echocardiography was performed using a Vivid ultrasound machine (GE Healthcare) with a Vivid Iq M4S‐RS Probe (GE Ving‐Med) connected to a 2.5‐ to 3.5‐MHz phased‐array probe. The patients were examined while at rest in the left lateral decubitus posture. According to the recommendations of the European Association of Cardiovascular Imaging,^11^ the apical four‐chamber, two‐chamber, and long‐axis images of three consecutive cycles were recorded using a standard high frame rate (>45 s^−1^).

Echocardiographic parameters included left atrial diameter, interventricular septum thickness, LV posterior wall thickness (PWTd) in the end‐diastolic period, which were obtained from 2D‐guided linear measurements. LV end‐diastolic volume (LVEDV), LV end‐systolic volume (LVESV) and LVEF were calculated from Teichholz's formula. Left atrial volume (LAV) was assessed utilizing the biplane method of disks at the average of the apical four‐chamber and two‐chamber. Transmitral Doppler was utilized to obtain the peak early (*E*) and late (*A*) diastolic mitral annular velocities. The peak early (*e*′) diastolic annular velocities were calculated by averaging the values at the septum and lateral wall using tissue Doppler imaging. Left ventricular mass (LVM) and relative wall thickness (RWT) were calculated using the standard formula based on recommendations from American Society of Echocardiography and the European Association of Cardiovascular Imaging.^11^ Left ventricular mass index (LVMI) and left atrial volume index (LAVI) were standardized to baseline BSA.

### 2.4 LV GLS and MW parameters

GLS and MW were evaluated on three consecutive cardiac cycles by Q‐analysis with EchoPAC software (EchoPAC Version 203, GE Healthcare). 2D‐STE collected data from three apical views (4, 2, and 3 chambers) at 50 and 80 frame/s. The gathered data were then used to assess the GLS of the LV myocardium. The operating doctors verified the software's automatic endocardial contour detection and adjusted the region of interest to ensure all images had the correct edge or width. MW parameter software included brachial artery BP, LV strain, and valve opening and closure timing data. Before an echocardiographic examination, the average of three seated BP readings were used to calculate the brachial artery BP. The left ventricle's GLS was determined before entering the patient's brachial artery BP. The software calculated four main parameters: global myocardial work index (GWI, mm Hg%), the annular area expressed as LV‐PSL, showed total work from mitral valve closure to opening. Global constructive work (GCW, mm Hg%), which benefited LV ejection, represented LV systolic shortening and diastolic lengthening. Global wasted work (GWW, mm Hg%), which was unfavorable to LV ejection, consisted of LV systolic stretching and diastolic shortening. Global myocardial work efficiency (GWE, %) was estimated using the formula GCW/(GCW + GCW), demonstrating the percentage of MW.

### 2.5 Intra‐ and Interobserver variability

Fifty randomly chosen participant images were remeasured by two skilled sonographers specializing in echocardiography. Sonographers were blinded to both the clinical data and each other's consequences during the process. The same sonographer re‐examined the images a month later to evaluate the intra‐observer variability. Both sonographers examined the identical images to evaluate Interobserver variability.

### 2.6 Statistical analysis

Continuous variables were expressed as mean ± SD or median with interquartile range and categorical data as absolute numbers and percentages. The Kolmogorov–Smirnov test evaluated continuous variables to assess the normality of distribution. Comparisons were made by independent samples *t*‐test for continuous variables and by chi‐square test or Mann–Whitney *U* test for categorical variables, as appropriate. There were three and four missing values on early (*E*) and late (*A*) diastolic mitral annular velocities, respectively, which were replaced with average values. Comparisons of groups (by phase and treatment) were performed using general linear models for repeated measures with one within‐subject two‐level factor (time points at phases 1 and 2) and one between‐subject two‐level factor (treatment groups). The Greenhouse–Geisser correction was used when the sphericity assumption, as assessed by Mauchly's test, was not met. If significant phase × treatment group interaction (*α* ≤ .10) was presented, separate analyses were conducted for each phase and treatment. General linear univariate analysis with prespecified covariates adjustment added in order was designed to evaluate the potential explanations for differences between treatments and the two‐phase points. Estimated marginal means analysis was performed with Bonferroni's correction. Univariate and multivariate linear regression analysis examined how intensive treatment affected LV systolic function alternation, as shown by MW parameter changes from phase 1 to phase 2. Clinical and echocardiographic variables were adjusted in a multivariate linear regression model: baseline age, sex, MW parameters in phase 1, ΔLVEDV, ΔLVESV, ΔGLS, ΔLVMI, ΔLAVI, Δ*E*/*e*′. The delta (Δ) indicates the changes from phase 1 to phase 2 as calculated by the variables in phase 2 minus those in phase 1. Interobserver and intraobserver agreement of MW parameters were assessed by the intraclass correlation coefficient (ICC) using two‐way random measures analysis and the Bland–Altman plot. The mean percentage error of each variable's two measurements was calculated. SPSS version 26.0 and the software GraphPad Prism 8.0 were performed to analyze all data. *p* < .05 was considered statistically significant.

## 3 RESULTS

### 3.1 Baseline characteristics

Sixty‐six participants were randomized to the intensive group and 50 to the standard treatment group. The median age was 66.0 (62.0–70.0) and 63.5 (61.0–69.0) years, respectively. The proportion of men in the intensive treatment group (42.4%) was larger than that in the standard treatment group (30.0%) without statistically significant difference. Smoking status was not statistically significant between the two groups (Table 1). Mean SBP was 140.2 ± 14.5 and 140.9 ± 12.7 mmHg at baseline, respectively. Both SBP distribution and the hypertension duration were not statistically different. Lipid profile, fasting serum glucose, and medication history between the two groups were similar without significant differences (Table 1).

**Table 1:** Baseline characteristics of the study population.

Characteristics	Intensive treatment (*N* = 66)	Standard treatment (*N* = 50)	*p* Value
Age (years), median (IQR)	66.0 (62.0–70.0)	63.5 (61.0–69.0)	.278
Male sex, *N* (%)	28 (42.4)	15 (30.0)	.170
Body‐mass index (kg/m^2^), median (IQR)	24.8 (23.2–27.2)	24.4 (22.4–24.3)	.181
Body surface area, m^2^	1.4 ± 0.5	1.6 ± 0.2	.064
Smoking status, *N* (%)			
Current smoker	6 (9.1)	5 (10.0)	.981
Former smoker	5 (5.1)	4 (8.0)	
Never smoker	55 (83.3)	41 (82.0)	
Heart rate, bpm	72 (67–78)	73 (66–77)	.592
Blood pressure, mm Hg			
Systolic	140.2 ± 14.5	140.9 ± 12.7	.791
Diastolic	83.0 ± 9.6	82.4 ± 8.4	.730
Distribution of systolic blood pressure, *N* (%)*			
≤135 mmHg	24 (36.4)	15 (30.0)	.770
136–147 mmHg	22 (33.3)	18 (36.0)	
≥148 mmHg	20 (30.3)	17 (34.0)	
Duration of hypertension, year	10.0 (5.0–15.0)	10.5 (6.0–16.0)	.543
Fasting serum glucose (mmol/L), median (IQR)	5.51 (5.10–6.50)	5.57 (5.01–6.60)	.804
Creatinine, mg/dL	0.83 (0.72–0.94)	0.79 (0.68–0.98)	.380
eGFR, mL/min per 1.73 m^2^	103.17 ± 21.42	104.00 ± 21.50	.836
Renal dysfunction, *N* (%)	2 (3.0%)	1 (2.0%)	.729
Lipid profile, mmol/L			
Total cholesterol	4.87 ± 1.10	5.08 ± 0.96	.291
Triglycetides, median (IQR)	1.28 (0.92–2.04)	1.46 (1.00–1.99)	.352
High‐density lipoprotein cholesterol, median (IQR)	1.14 (1.01–1.47)	1.20 (1.07–1.49)	.254
Low‐density lipoprotein cholesterol	2.69 ± 0.86	2.69 ± 0.81	.995
Comorbidity, *N* (%)			
Diabetes mellitus	14 (21.2)	15 (30.6)	.269
Hyperlipidemia	27 (40.9)	24 (49)	.565
Cardiovascular disease, *N* (%)	5 (7.6)	4 (8.0)	.933
Medication history, *N* (%)			
Use of antihypertensive agents	65 (98.5)	49 (98.0)	.843
Use of hypoglycemic agents	11 (16.7)	15 (30.0)	.088
Use of lipid‐lowering drugs	19 (28.8)	16 (32.0)	.709
Use of antiplatelet drugs	7 (10.6)	5 (10.0)	.915
No. of antihypertensive agents	1.6 ± 0.6	1.4 ± 0.6	.088

*p < .05 was considered statistically significant.

Abbreviations: IQR, interquartile range.

### 3.2 BP and conventional echocardiographic findings

SBP presented a statistically significant interaction between treatment and phase (interaction *p* = .017). In phase 1 echocardiographic examination, SBP was already significantly lower in the intensive treatment group than the standard treatment group (126.5 vs. 132.1 mmHg, *p* < .05). Following a further 2‐year treatment period, the intensive group continued to exhibit a lower SBP compared to the standard treatment group (125.0 vs. 135.3 mmHg, *p* < .05) (Table 2). In the intensive treatment group, there was no statistically significant difference observed in SBP between phase 1 and phase 2 (126.5 vs. 125 mmHg, *p* > .05). In the standard treatment group, there was a statistically significant increase in SBP during phase 2 compared to phase 1 (132.1 vs. 135.3 mmHg, *p* < .05).

**Table 2:** Blood pressure and conventional echocardiographic parameters between two groups and study phase.

	Intensive treatment (*N* = 66)		Standard treatment (*N* = 50)		*p* Value	
	Phase 1	Phase 2	Phase 1	Phase 2	Treatment	Time
Blood pressure						
SBP (mmHg)	126.5 ± 9.0	125.0 ± 7.7	132.1 ± 9.2	135.3 ± 7.3	.017† ^,^ ‡ ^,^ §	
DBP (mmHg)	75.2 ± 5.6	76.3 ± 7.2	77.5 ± 7.7	78.7 ± 8.2	.039	.129
Conventional echocardiographic parameters						
LAD (cm)	3.43 ± 0.41	3.35 ± 0.39	3.32 ± 0.38	3.22 ± 0.38	.006§	
IVSd (cm)	0.99 ± 0.13	0.98 ± 0.13	0.98 ± 0.11	0.95 ± 0.11	.282	.066
PWTd (cm)	0.94 ± 0.14	0.97 ± 0.13	0.90 ± 0.11	0.95 ± 0.11	.152	.001
LVEDV (mL)	106.68 ± 22.99	99.68 ± 22.86	93.66 ± 17.58	91.30 ± 21.51	.005	.004
LVESV (mL)	33.17 ± 8.96	32.91 ± 11.27	28.92 ± 6.46	29.10 ± 11.27	.011	.968
LVEF (%)	68.98 ± 3.87	67.76 ± 4.89	68.84 ± 3.63	67.62 ± 5.77	.827	.035
E/A ratio	0.77 ± 0.17	0.72 ± 0.18	0.81 ± 0.20	0.76 ± 0.22	.208	.008
LAVI (mL/m^2^)	33.07 ± 8.79	26.58 ± 6.68	30.74 ± 7.38	27.07 ± 6.84	.585	<.001
Mean *e*′ (cm/s)	7.18 ± 1.49	7.08 ± 1.88	7.48 ± 1.52	7.16 ± 1.87	.504	.173
Mean *E*/*e*′	10.64 ± 3.19	9.50 ± 2.91	10.49 ± 2.56	10.00 ± 3.19	.709	.003
LVMI (g/m^2^)	93.30 ± 25.95	94.50 ± 25.55	101.62 ± 24.69	99.26 ± 24.40	.141	.737
RWT	0.40 ± 0.01	0.43 ± 0.01	0.40 ± 0.01	0.43 ± 0.01	.476	<.001

*Note*: If no interaction between phase and treatment group exists, then data were analyzed together, with the *p* values as shown in the final two columns. If a phase × treatment interaction exists (i.e., effects differ over time between the two groups), this *p* value is shown across both final columns, then data were analyzed separately by phase and treatment. *p* < .05 phase 1 versus phase 2 intensive.

Abbreviations: *A*, A wave; DBP, diastolic blood pressure; *E*, E wave; *e*′, early diastolic velocity; IVSd, interventricular septal thickness in diastole; LAD, left atrial diameter; LAV, left atrial volume; LVEDD, left ventricular end‐diastolic dimension; LVEDV, left ventricular end diastolic volume; LVEF, left ventricular ejection fraction; LVESD, left ventricular end‐systolic dimension; LVESV, left ventricular end systolic volume; LVM, left ventricular mass; LVMI, left ventricular mass index, RWT, relative wall thickness; PWTd, posterior wall thickness in diastole; SBP, systolic blood pressure.

^†^ *p* < .05 intensive versus standard phase 1.

^‡^ *p* < .05 intensive versus standard phase 2.

^§^ *p* < .05 phase 1 versus phase 2 standard.

LVEDV decreased from phase 1 to phase 2 in both groups, with a significant treatment difference (*p* < .05). From phase 1 to phase 2, PWTd and RWT increased in both groups, without significant differences between the treatments. Besides, LAVI, E/A, and *E*/*e*′ did not differ significantly across both treatments; however, they all declined from phase 1 to phase 2 in both groups. Both treatment groups had slightly reduced LVEF maintaining in normal, but the treatment effects did not differ significantly (Table 2).

### 3.3 GLS and MW analysis

**Figure 1:**
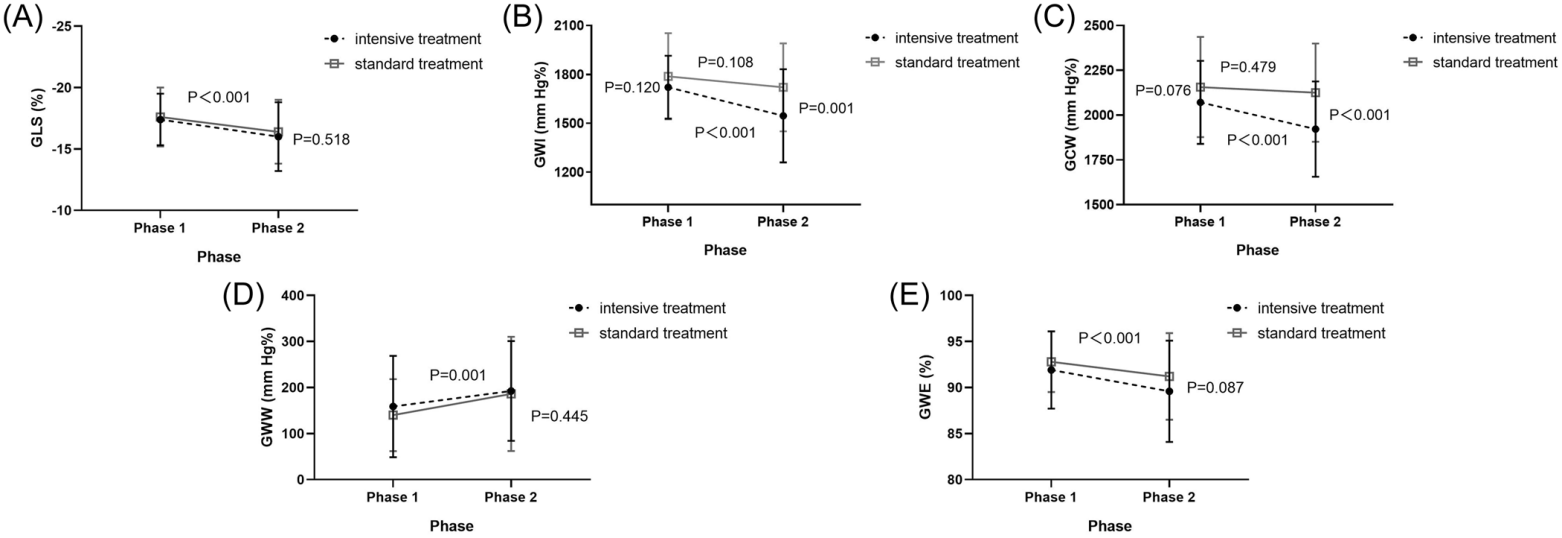
Impact of two treatment groups at phase 1 and 2 on GLS (A), GWI (B), GCW (C), GWW (D), and GWE (E). For GLS, GWW, and GWE, there was no significant treatment × phase interaction so there was only a single *p* value for the change with phase and the change with treatment. The other two parameters including GWI and GCW exhibited a significant treatment × phase interaction, and so there were *p* values for the treatment group at phase 1 and phase 2 and for the change with phase for both groups separately. GCW, global constructive work; GWE, global work efficiency; GWI, global work index; GWW, global wasted work.

The analysis revealed a statistically significant phase×time interaction for GWI (*p* = .052). Specifically, the intensive treatment group exhibited a significant decrease in GWI from phase 1 to phase 2 (*p* < .001), whereas the standard treatment group did not show any significant change in GWI after a 2‐year follow‐up (*p* = .108). Furthermore, GWI in the intensive treatment group was found to be similar to that of the standard treatment group in phase 1 (*p* = .120), but significantly lower in phase 2 (*p* = .001). GCW also had a phase × time interaction (*p* = .045). The intensive treatment group's GCW decreased from phase 1 to phase 2 (*p* < .001), and the standard treatment group displayed unchanged GCW (*p* = .479). GCW in the intensive group was comparable with the intensive group in phase 1 (*p* = .076), but was significantly lower than the standard group in phase 2 (*p* < .001). The GWW showed a time effect (*p* = .001) with an increase in both groups over a period of 2 years, and there was no statistically significant difference observed in the treatment effect (*p* = .445). Both the GLS and GWE demonstrated a significant time effect (*p* all <.001) as both groups exhibited a decrease in performance over a 2‐year period. Furthermore, no statistically significant difference was seen in the treatment effect (*p* > .05) (Table 3 and Figure 1). We investigated whether potential confounding factors could explain the differences in GLS, GWI, GCW, GWW, and GWE between the two treatment groups, using general linear model, added age and sex, BSA, SBP and DBP, HR, and phase 1 GLS in order. The estimated marginal means results were similar to the unadjusted analysis (Supporting Information: Table S2) for GLS and all MW parameters in phase 2.

**Table 3:** GLS and myocardial work parameters between two groups and study phase.

	Intensive treatment (*N* = 66)		Standard treatment (*N* = 50)		*p* Value	
	Phase 1	Phase 2	Phase 1	Phase 2	Treatment	Phase
GLS (%)	−17.4 ± 2.1	−16.0 ± 2.8	−17.6 ± 2.4	−16.4 ± 2.6	.518	<.001
GWI (mmHg%)	1721.4 ± 192.8	1546.0 ± 286.1	1788.0 ± 264.4	1720.6 ± 269.7	.052*, ^‡^	
GCW (mmHg%)	2071.4 ± 231.5	1922.4 ± 265.9	2156.5 ± 279.8	2125.3 ± 274.2	.045*, ^‡^	
GWW (mmHg%)	158.7 ± 110.2	192.4 ± 108.3	140.0 ± 78.1	186.1 ± 124.0	.445	.001
GWE (%)	91.9 ± 4.2	89.6 ± 5.5	92.8 ± 3.3	91.2 ± 4.7	.087	<.001

*Note*: If no interaction between phase and treatment group exists, then data were analyzed together, with the *p* values as shown in the final two columns. If a phase × treatment interaction exists (i.e., effects differ over time between the two groups), this *p* value is shown across both final columns, then data were analyzed separately by phase and treatment. *p* < .05 intensive versus standard phase 1. *p* < .05 phase 1 versus phase 2 standard.

Abbreviation: GCW, global constructive work; GLS, global longitudinal strain; GWE, global work efficiency; GWI, global work index; GWW, global wasted work.

* *p* < .05 phase 1 versus phase 2 intensive.

‡ *p* < .05 intensive versus standard phase 2.

### 3.4 Regression analyses of the changes in GWI and GCW

Univariate linear regression analysis showed the intensive treatment was significantly associated with ΔGCW (*β* = −117.80; 95% CI, −232.96 to −2.63, *p* = .045) but not with ΔGWI (*β* = −108.01; 95% CI, −217.14 to 1.108, *p* = .052). After adjusting for baseline age, sex, phase 1 GWI, ΔLVEDV, ΔLVESV, ΔGLS, ΔLVMI, ΔLAVI, and Δ*E*/*e*′, the multivariable linear regression model revealed a statistically significant relationship between intensive treatment and ΔGWI (*β* = −110.92; 95% CI, −197.78 to −30.07, *p* = .008). Intensive treatment presented statistically significant for ΔGCW in multivariable linear regression analysis after adjusting covariates (*β* = −477.76; 95% CI, −234.53 to −60.98, *p* = .001) (Table 4). Higher phase 1 GWI was significantly correlated with a more decline in GWI from 1 to 2 phase (*β* = −0.39; 95% CI, −0.58 to −0.21, *p* < .001). Similarly, the GCW value in phase 1 was a significant independent predictor of ΔGCW (*β* = −0.60; 95% CI, −0.77 to −0.42, *p* < .001). After controlling for variables, ΔGLS was determined to be significantly correlated with both ΔGWI (*β* = −57.93; 95% CI, −73.85 to −42.01, *p* < .001) and ΔGCW (*β* = −45.99; 95% CI, −63.14 to −28.83, *p* < .001). Besides, the ΔLVEDV increased with increasing ΔGWI (*β* = 2.61; 95% CI, 0.24 to 4.97, *p* = .031) in a positive relationship. Sex was also revealed as an independent predictor of ΔGCW (−95.51; 95% CI, −183.69 to −7.34) (Table 4).

**Table 4:** Univariate and multivariate linear regression analysis showing associations of clinical variables and the change of echocardiographic variables with the change of global work index and global constructive work.

	Univariate analysis		Multivariate analysis	
	*β* (95% CI)	*p* Value	*β* (95% CI)	*p* Value
ΔGWI				
Treatment	−108.01 (−217.14 to 1.108)	.052	−110.92 (−191.78 to −30.07)	.008
Baseline age	−10.03 (−21.42 to 1.36)	.084		
Sex	−0.122 (−187.78 to 38.03)	.192		
Phase 1 GWI	−0.54 (−0.76 to −0.31)	<.001	−0.39 (−0.58 to −0.21)	<.001
ΔLVEDV	5.49 (2.46 to 8.53)	<.001	2.61 (0.24 to 4.97)	.031
ΔLVESV	4.90 (− 0.48 to 10.27)	0.74		
ΔGLS	−70.01 (−87.120 to −52.90)	<.001	−57.93 (−73.85 to −42.01)	<.001
ΔLVMI	−1.00 (−4.00 to 2.00)	.511		
ΔLAVI	3.74 (−2.95 to 10.43)	.270		
Δ*E*/*e*′	−11.31 (−30.26 to 7.64)	.240		
ΔGCW				
Treatment	−117.80 (−232.96 to −2.63)	.045	−147.76 (−234.53 to −60.98)	.001
Baseline age	−9.74 (−21.80 to 2.32)	.112		
Sex	−0.12 (−197.29 to 41.33)	.198	−95.51 (−183.69 to −7.34)	.034
Phase 1 GCW	−0.63 (−0.823 to −0.431)	<.001	−0.60 (−0.77 to −0.42)	<.001
ΔLVEDV	5.27 (2.04 to 8.51)	.002		
ΔLVESV	2.11 (−3.63 to 7.86)	.468		
ΔGLS	−59.76 (−79.56 to −39.95)	<.001	−45.55 (−62.44 to −28.65)	<.001
ΔLVMI	−0.21 (−3.38 to 2.97)	.898		
ΔLAVI	0.91 (−6.19 to 8.012)	.800		
Δ*E*/*e*′	−9.34 (−29.41 to 10.73)	.359		

*Note*: Δ indicates change from phase 1 to phase 2, phase 2 minus phase 1.

Abbreviation: *E*, E wave; *e*′, early diastolic velocity; GCW, global constructive work; GLS, global longitudinal strain; GWI, global work index; LA, left atrial; LVEDV, left ventricular end diastolic volume; LVESV, left ventricular end systolic volume; LVM, left ventricular mass.

### 3.5 Evaluation of intra‐ and interobserver variabilities

The Bland–Altman plots and ICC analysis of inter‐ and intraobserver agreement are shown in Supporting Information: Figure S2 and Table S3.

## 4 DISCUSSION

We first examined the association of MW parameters and intensive BP treatment. Our findings indicated a significant reduction in GWI and GCW from phase 1 to phase 2 in the intensive treatment group. The standard treatment group exhibited no significant alterations in GWI and GCW. Intensive treatment was also independently correlated with ΔGWI and ΔGCW. Both groups revealed no significant difference in treatment effects in terms of LVEF and GLS. Therefore, GWI and GCW were more sensitive to the changes during intensive BP treatment than GLS and LVEF. Besides, we also discovered GWW increased and GWE decreased in the two treatment groups from phase 1 to phase 2.

Few research examined BP‐lowering treatment mechanisms with MW parameters. The majority of studies examining the effects of treatment on MW parameters utilized prospective, self‐control, before‐and‐after methodologies. In contrast, our research employed a randomized, controlled approach in the subcenter STEP trial. Before‐and‐after treatment effect on sacubitril/valsartan was tested in hemodialysis patients with resistant hypertension using MW parameters.^12^ A randomized controlled study evaluated the cardiac effects of insulin, glucagon‐like peptide‐1 receptor agonists (GLP‐1RA), sodium‐glucose cotransporter‐2 inhibitors (SGLT‐2i), and GLP‐1RA + SGLT‐2i on diabetics.^13^ The STRUCTURE trial demonstrated GCW could quantify LV contractile response to exertion in heart failure with preserved ejection fraction (HFpEF) patients treated for spironolactone (6 months) compared to placebo, which paralleled the role of GCW in our findings.^14^ Nevertheless, our study population, intervention strategies, research purpose, and follow‐up duration varied considerably from those of the aforementioned two randomized controlled studies. Thus, to our knowledge, the present study is the first prospective assessment of intensive treatment with an SBP goal of 110 to <130 mmHg utilizing MW to evaluate LV systolic function in the 60 to 80‐year‐old hypertensives.

Subclinical LV systolic dysfunction is especially important for clinical prognosis in hypertensive patients with normal LVEF. Among patients without heart failure (HF) at baseline, impaired systolic function based on LVEF or strain assessment was independently correlated with incident HF, acute myocardial infarction, or cardiovascular death including elderly population.^4^, ^15^ LVEF evaluates left ventricular systolic function regularly in clinical practice, but the assessment is extremely complex and cannot be adequately determined by LVEF alone.^16^ GLS is recommended by the American Society of Echocardiography and the European Society of Cardiovascular Imaging^17^ as a more accurate predictor of cardiovascular outcomes than LVEF.^18^ However, previous studies demonstrated that afterload may decrease GLS, but not directly point to myocardial contractility reduction.^19^, ^20^ MW technique is superior to LVEF and GLS for eliminating the influence of afterload on cardiac function assessment.^21^ Of importance, previous studies found MW parameters were more sensitive than LVEF and GLS in detecting subclinical LV systolic dysfunction. Our research showed LVEF and GLS in both treatment groups without difference, which proved that MW was better than GLS and LVEF in identifying subtle systolic function changes between the two different treatments in the elderly hypertensive patients.

A meta‐analysis research included 1140 hypertensive patients (mean age 55.4 years, 50% men, follow‐up 6–36 months) containing 8 studies demonstrated that antihypertensive treatment was associated with the improvement in GLS.^22^ However, we found both groups with stable SBP control decreased GLS from phase 1 to 2 without significant difference between treatment effects. Meanwhile, no association was found between SBP reduction and GLS improvement in the above study.^22^ This equivocal result could be explained by cardiac interstitial fibrosis, which develops in HHD and is associated with reduced GLS.^23^ GLS was proved to be an accurate noninvasive indicator of cardiac interstitial fibrosis in animal experiments.^24^, ^25^ Heart fibrosis is a common consequence of endothelial dysfunction caused by inflammation and oxidative stress in aging, hypertension, diabetes mellitus, and obesity, eventually leading to cardiovascular disease, heart failure, and CKD.^26^ Therefore, the results seemed to indicate that adequate controlled SBP did not appear to prevent the progression of myocardial interstitial fibrosis in elderly hypertensive patients.

In a retrospective cross‐sectional study by Tadic et al.^27^ GWI progressed steadily from healthy controls (average SBP: 126 mmHg) through controlled (average SBP: 129 mmHg) and uncontrolled (average SBP: 148 mmHg) patients, to hypertensive patients with resistant hypertension (average SBP: 147 mmHg). GCW showed the same results. Similar research results were also presented in a single‐center, retrospective study.^28^ The Stage 1 hypertension group (SBP: 134.5 ± 3.6 mmHg) had higher GWI and GCW than non‐hypertensive control (SBP: 113.5 ± 11.9 mmHg), but a significant difference did not present in Stage 1 and Stage 2 (SBP: 156.2 ± 8.8 mmHg) hypertension group.^28^ Another similar research revealed moderate‐to‐severe hypertension patients (SBP: ≥160 mmHg) had higher GWI and GCW values in comparison to mild hypertension patients (SBP: 140‐159 mmHg) with a significant difference.^29^ That may illustrate in the standard treatment group, higher LV systolic pressure causes an increase in afterload, requiring the LV to do more effort to complete mechanical systole.^28^ Hence, the work done by the myocardium due to the compensatory effect will decrease in the intensive treatment group. Taken together, it may explain why intensive treatment patients had a significant decrease in GWI and GCW from phase 1 to phase 2, but not appeared in the standard treatment group. Besides, as the baseline SBP value was comparable in both groups, cardiac compensatory work of the intensive treatment group might be not yet changed obviously in 1‐year follow‐up. Therefore, GWI and GCW did not differ after the first phase's treatment between the two groups. After nearly 3‐year long‐term anti‐hypertensive treatments with well‐targeted SBP control in phase 2, the intensive group had much lower GWI and GCW than the standard group significantly. It is noteworthy that both groups, regardless of whether they were in phase 1 or phase 2, had median values of GWI and GCW that fell within the normal range as determined by the Copenhagen City Heart Study (CCHS).^30^ Hospitalization for myocardial infarction (MI) within the last 6 months, coronary revascularization or bypass grafting within the last 12 months or planned in the next 12 months, New York Heart Association (NYHA) class III–IV heart failure, or exacerbation of chronic heart failure at entry were excluded at baseline from our study so that the heart blood supply in this study population was relatively normal. Therefore, a decrease in GWI and GCW did not necessarily indicated a decrease in cardiac systolic function. Furthermore, sex differences in the effect of anti‐hypertensive treatment were observed,^31^ which could explain the discrepancies of ΔGCW in our study. The results partially reflected the effect of antihypertensive treatment on gender disparities in LV systolic function changes.

Ding et al. found that GWW was higher in the mild hypertension group (SBP: 140–159 mmHg) and moderate‐to‐severe hypertension group (SBP: ≥160 mmHg) than the control group (SBP: 119.3  ± 10.4 mmHg).^29^ Our study discovered that GWW increased from phase 1 to phase 2 in both groups, but there was no significant difference in treatment effect. The increase in GWW may have to do with the fact that hypertensive patients who had higher levels of afterload for an extended period and had stiffer myocardial walls.^29^ Jaglan's research results also supported the findings on GWW.^28^ The elevation in GWW may result from LV fibrosis and stiffness brought on by persistent hypertension.^32^ Our study also demonstrated GWE reduced significantly in both groups without treatment effect difference. The increase in GWW and decrease in GWE in both groups were consistent with the decrease in LVEF and GLS from our study. It was acknowledged that despite proper control of BP, certain individuals with hypertension continued to experience cardiac fibrosis and microvascular atherosclerosis, which posed challenges in terms of achieving complete reversal. The aforementioned minor alterations, which resulted in a reduction in the supply of blood and oxygen to the myocardium, could potentially result in disorganized myocardial contractions, hence causing an increase in GWW and a decrease in GWE. The Copenhagen City Heart Study (CCHS) explored MW parameters normal value in 1827 participants, which discovered that GWW and GWE were influenced by age and gender‐specific differences.^30^ GWE decreased with age‐related increases in GWW, especially for women.^30^ CCHS study determined GWE was calculated by comparing GWW to GCW; thus, any change in GWE presumably reflects the natural rise in GWW that occurs with age.^30^ Truong et al. investigated that decreased GWE was associated with aging in meta‐analysis.^33^ Other comorbidities in both groups such as diabetes, insulin resistance, overweight, obesity, dyslipidemia, and subclinical coronary artery diseases may also played a significant role in endothelial dysfunction and the development of atherosclerosis, which contributed to the increase in GWW and the decline in GWE.^6^

## 5 LIMITATIONS

This study's limitations need to be discussed. Our phase 2 data were insufficient to examine cardiovascular events and mortality. Therefore, we could not determine the MW parameters' prognostic threshold values, which prevented us from demonstrating how MW parameters would affect the study's endpoint. Otherwise, no echocardiographic data were collected pre‐randomization. Additionally, we lack laboratory test results for further repeated analysis. The slightly poor quality of echocardiographic images may affect how the LV mechanics and MW were evaluated during echocardiography. Clinical research was unavoidable and did not introduce relevant bias. This study measured MW parameters utilizing clinic‐visit SBP rather than supine BP recorded during the echocardiography. The MW technique used the brachial artery BP rather than the LV wall stress to estimate LV pressure, ignoring the effect of the LV's absolute size and the wall curvature.^34^, ^35^

## 6 CONCLUSIONS

Our study demonstrated that MW parameters were more sensitive to the changes of systolic function during intensive BP treatment than traditional parameters. Furthermore, intensive BP treatment was associated with reduced GWI and GCW in the elderly hypertensive patients. GWW increased and GWE declined in the both groups. We demonstrated that intensive antihypertensive treatment did not reverse MW in elderly hypertensive patients. Further research of our group should pay more attention to determine the prognostic value of MW on cardiovascular outcome during the hypertension treatment.

## AUTHOR CONTRIBUTIONS

All authors have made an important scientific contribution to the study. Yingqing Feng and Linghui Tang participated in study design. Xiaoxuan Feng, Mengqi Yan, Shiping Wu, and Yingqing Feng contributed to data analysis and interpretation. Xiaoxuan Feng, Mengqi Yan, Linghui Tang, Dan Zhou, and Yingqing Feng were responsible for drafting the manuscript or revising it critically for important intellectual content. All authors listed have read and approved submission of the paper.

## CONFLICT OF INTEREST STATEMENT

The authors declare no conflict of interest.

## Supporting information

Supporting information.

Supporting information.

Supporting information.

## Data Availability

All data relevant to the study are included in the article. The datasets used and/or analyzed during this investigation are available upon reasonable request from the corresponding author
