# Supplementary material for: Effects of intensive blood‐pressure treatment on myocardial work in elderly hypertensive patients: A subcenter study of the STEP randomized controlled trial
Source: Clin Cardiol. 2023 Oct 11;47(1):e24172. doi: 10.1002/clc.24172 (PMC10766135; doi:10.1002/clc.24172)
Supplement: Supplementary file 1 — Supporting information. [file CLC-47-e24172-s003.docx]

**SUPPLEMENTARY**

**Supplemental Methods**

Fomula:

eGFR = 186 × (Creatine / 88.4)^-1.154^ × (Age)^-0.203^ × 1.227 × (0.742 if female).

LVM = 0.8 [1.04 (IVSd+LVEDD+PWTd)^3^ – (LVEDD)^3^] + 0.6 g

RWT = 2×PWTd / LVEDD

Teichholz’s formula:

LVEDV = (7 / (2.4 +LVEDD)) × (LVEDD^3^)

LVESV = (7 / (2.4 +LVEDD)) × (LVEDD^3^)

LVEF = (LVEDV–LVESV / LVEDV) × 100%

|  | **STEP cohort** | | **Substudy cohort** | |
| --- | --- | --- | --- | --- |
| **Characteristics** | **Intensive group**  **(N=4243)** | **Standard group**  **(N=4268)** | **Intensive group**  **(N=66)** | **Standard group**  **(N=50)** |
| Age, y | 66.2 ± 4.8 | 66.3 ± 4.8 | 66.0 (62.0-70.0) | 63.5 (61.0-69.0) |
| Male sex, N (%) | 1990 (46.9) | 1969 (46.1) | 28 (42.4) | 15 (30.0)* |
| Body-mass index, kg/m^2^ | 25.5 ± 3.2 | 25.6 ± 3.2 | 24.8 (23.2-27.2) | 24.4 (22.4-24.3)* |
| Systolic blood pressure, mmHg | 146.1 ± 16.8 | 146.0 ± 16.5 | 140.2 ± 14.5* | 140.9 ± 12.7* |
| Diastolic blood pressure, mmHg | 82.7 ± 10.6 | 82.3 ± 10.5 | 83.0 ± 9.6 | 82.4 ± 8.4 |
| Diabetes melitus, N (%) | 800 (18.9) | 827 (19.4) | 14 (21.2) | 15 (30.6) |
| History of cardiovascular disease, N (%) | 268 (6.3) | 272 (6.4) | 5 (7.6) | 4 (8.0) |
| Total cholesterol, mmol/L | 4.9 ± 1.2 | 4.9 ± 1.1 | 4.87 ± 1.10 | 5.08 ± 0.96 |
| Triglycetides, median, mmol/L | 1.3 (1.0-2.0) | 1.4 (1.0-1.9) | 1.28 (0.92-2.04) | 1.46 (1.00-1.99) |
| High-density lipoprotein cholesterol, mmol/L | 1.3 ± 0.3 | 1.3 ± 0.3 | 1.14 (1.01-1.47)* | 1.20 (1.07-1.49) |
| Low-density lipoprotein cholesterol, mmol/L | 2.7 ± 0.9 | 2.7 ± 0.9 | 2.69 ± 0.86 | 2.69 ± 0.81 |
| Fasting serum glucose, mmol/L | 6.2 ± 1.8 | 6.2 ± 1.7 | 5.51 (5.10-6.50) | 5.57 (5.01-6.60)* |

**Table S1 Baseline characteristics for the full STEP cohort (N=8511) and the Substud cohort in the current study (N=116). Differences between the full and smaller cohorts by treatment are identified (*). Continuous variables were summarized as mean (SD) or medians (quartile interval) and categorical variables were displayed as percentage (%)**

Abbreviation: STEP, Strategy of Blood Pressure Intervention in the Elderly Hypertensive Patients Trial.

**Table S2 Multivariate analysis of GLS and Myocadial work Incdice between groups in phase 2**

| **Echocardiographic  characteristic** | **Intensive Treatment (N=66)** | **Standard Treament (N=50)** | **P value** |
| --- | --- | --- | --- |
| **GLS (%)** | | | |
| Model 1 adjusted for age, sex | -16.0 ± 0.3 | -16.3 ± 0.4 | 0.650 |
| Model 2 adjusted for model 1, BSA | -16.2 ± 0.3 | -16.3 ± 0.4 | 0.857 |
| Model 3 adjusted for model 2, SBP, DBP | -16.2 ± 0.3 | -16.3 ± 0.4 | 0.840 |
| Model 4 adjusted for model 3, HR | -16.2 ± 0.3 | -16.2 ± 0.4 | 0.905 |
| Model 5 adjusted for model 4, Phase 1 GLS | -16.2 ± 0.3 | -16.3 ± 0.3 | 0.815 |
| **GWI (mm Hg%)** | | | |
| Model 1 adjusted for age, sex | 1555.0 ± 33.9 | 1708.7 ± 39.0 | 0.004 |
| Model 2 adjusted for model 1, BSA | 1558.5 ± 34.1 | 1704.1 ± 39.3 | 0.007 |
| Model 3 adjusted for model 2, SBP, DBP | 1557.8 ± 34.3 | 1705.1 ± 39.5 | 0.006 |
| Model 4 adjusted for model 3, HR | 1560.8 ± 34.1 | 1701.0 ± 39.4 | 0.009 |
| Model 5 adjusted for model 4, Phase 1 GWI | 1572.1 ± 31.9 | 1686.1 ± 36.9 | 0.024 |
| **GCW (mm Hg%)** | | | |
| Model 1 adjusted for age, sex | 1930.8 ± 32.7 | 2114.3 ± 37.7 | <0.001 |
| Model 2 adjusted for model 1, BSA | 1938.7 ± 32.3 | 2103.9 ± 37.2 | 0.001 |
| Model 3 adjusted for model 2, SBP, DBP | 1938.5 ± 32.0 | 2104.2 ± 37.6 | 0.001 |
| Model 4 adjusted for model 3, HR | 1942.8 ± 32.0 | 2098.5 ± 36.9 | 0.002 |
| Model 5 adjusted for model 4, Phase 1 GCW | 1954.4 ± 32.0 | 2083.2 ± 35.2 | 0.008 |
| **GWW (mm Hg%)** | | | |
| Model 1 adjusted for age, sex | 192.7 ± 14.1 | 185.7 ± 16.2 | 0.744 |
| Model 2 adjusted for model 1, BSA | 192.8 ± 14.2 | 185.6 ± 16.4 | 0.746 |
| Model 3 adjusted for model 2, SBP, DBP | 194.1 ± 14.1 | 183.8 ± 16.3 | 0.697 |
| Model 4 adjusted for model 3, HR | 194.1 ± 14.1 | 183.8 ± 16.3 | 0.639 |
| Model 5 adjusted for model 4, Phase 1 GWW | 191.6 ± 13.6 | 187.1 ± 15.7 | 0.832 |
| **GWE (%)** | | | |
| Model 1 adjusted for age, sex | 89.6 ± 0.6 | 91.1 ± 0.7 | 0.117 |
| Model 2 adjusted for model 1, BSA | 89.7 ± 0.6 | 91.1 ± 0.7 | 0.159 |
| Model 3 adjusted for model 2, SBP, DBP | 89.7 ± 0.6 | 91.1 ± 0.7 | 0.146 |
| Model 4 adjusted for model 3, HR | 89.7 ± 0.6 | 91.1 ± 0.7 | 0.149 |
| Model 5 adjusted for model 4, Phase 1 GWE | 89.9 ± 0.6 | 90.8 ± 0.7 | 0.289 |

Abbreviation: GLS, global longitudinal strain; GWI, global work index; GCW, global constructive work; GWW, global wasted work; GWE, global work efficiency; BSA, body surface area; SBP, systolic blood pressure; DBP, diastolic blood pressure; HR, heart rate.

**Table S3 Intraclass correlation coefficient (ICC) analysis for Inter-observer and intra-observer repeatability and reproducibility of MW parameters**

|  | **Inter-observer reliability** | | | **Intra-observer reliability** | | |
| --- | --- | --- | --- | --- | --- | --- |
|  | **Bias, mean**  **(SD)** | **ICC**  **(95% CI)** | **Mean percentage error, % (SD)** | **Bias, mean**  **(SD)** | **ICC**  **(95% CI)** | **Mean percentage error, % (SD)** |
| GLS (%) | 1.04  (1.34) | 0.757 (0.361-0.891) | 5.71  (7.39) | -0.10  (1.04) | 0.901  (0.833-0.943) | 0.01  (0.01) |
| GWI (mm Hg%) | -82.28  (153.80) | 0.733 (0.494-0.856) | 4.31  (9.07) | 29.28  (123.1) | 0.867  (0.776-0.922) | 0.01  (0.07) |
| GCW (mm Hg%) | -105.78  (181.53) | 0.693 (0.411-0.836) | 4.43  (8.39) | 40.00  (141.80) | 0.863  (0.770-0.920) | 0.02  (0.06) |
| GWW (mm Hg%) | 4.12  (73.97) | 0.758 (0.609-0.855) | 11.40  (51.07) | 7.54  (52.12) | 0.863  (0.771-0.920) | -0.01  (0.29) |
| GWE (%) | -0.92  (2.91) | 0.796 (0.659-0.880) | 1.00  (3.26) | 0.10  (2.47) | 0.828  (0.716-0.899) | 0.00  (0.03) |

ICC represent the between-pairs variance expressed as a proportion of the total variance of the observations, where ICC ranges from 0 to 1, and values > 0.6 were considered to represent substantial reliability. Investigating inter-observer variation, the ICC was 0.757, 0.733, 0.693, 0.758, 0.796 for GLS, GWI, GCW, GWW and GWE, respectively (P all <0.05), and overall mean percentage error in the calculation was 5.37% for the efficacy measures. Regarding inter-observer variation, the ICC was 0.901, 0.867, 0.863, 0.863, 0.828 for GLS, GWI, GCW, GWW, and GWE, respectively (P all <0.05), and the overall mean percentage error was 0.006%.

Abbreviation: GLS, global longitudinal strain; GWI, global work index; GCW, global constructive work; GWW, global wasted work; GWE, global work efficiency; SD, standard deviation, CI, confidence interval; ICC, intraclass correlation coefficient.

**Figure S1.** Study Flow Chart.

**Figure S2.** Intra-observer (A1-E1) and Inter-observer (A2-E2) agreement of Bland-Altman plot for GLS (A1, A2), GWI (B1, B2), GCW (C1, C2), GWW (D1, D2), GWE (E1, E2) repeatability and reproductivity of Myocardial Work Parameters. Red dashed lines represent average agreement and the black dashed lines represent 95% CI limit of agreement. The Bland-Altman plots of inter- and intra-observer agreement demonstrated no proportionate bias for GLS, GWI, GCW, GWW, and GWE.

Abbreviation: GLS, global longitudinal strain; GWI, global work index; GCW, global constructive work; GWW, global wasted work; GWE, global work efficiency.
