# Supplementary figures and images for: Effects of intensive blood‐pressure treatment on myocardial work in elderly hypertensive patients: A subcenter study of the STEP randomized controlled trial
Source: Clin Cardiol. 2023 Oct 11;47(1):e24172. doi: 10.1002/clc.24172 (PMC10766135; doi:10.1002/clc.24172)

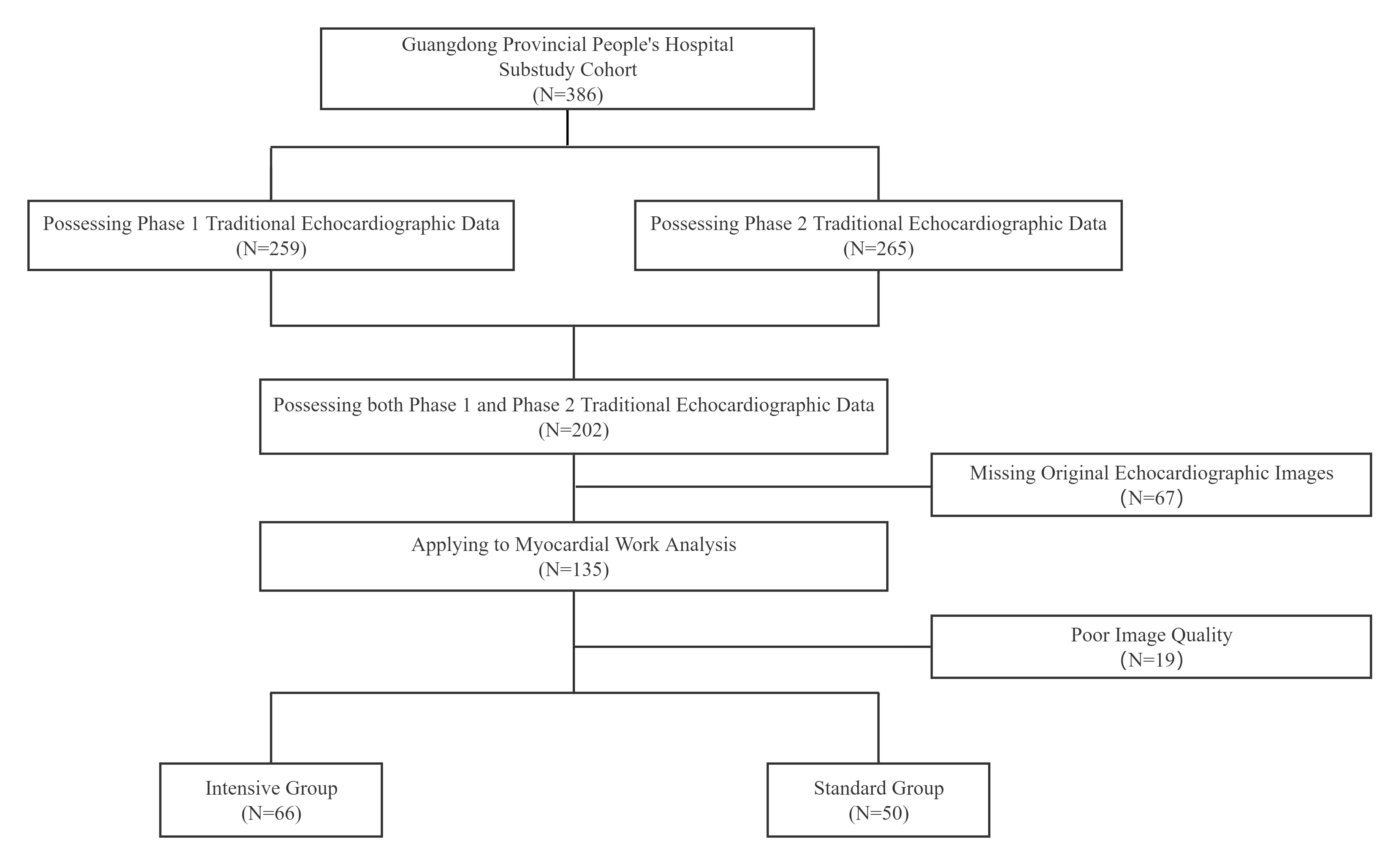

Supplement: Supplementary file 2 — Supporting information. [file CLC-47-e24172-s002.jpg]

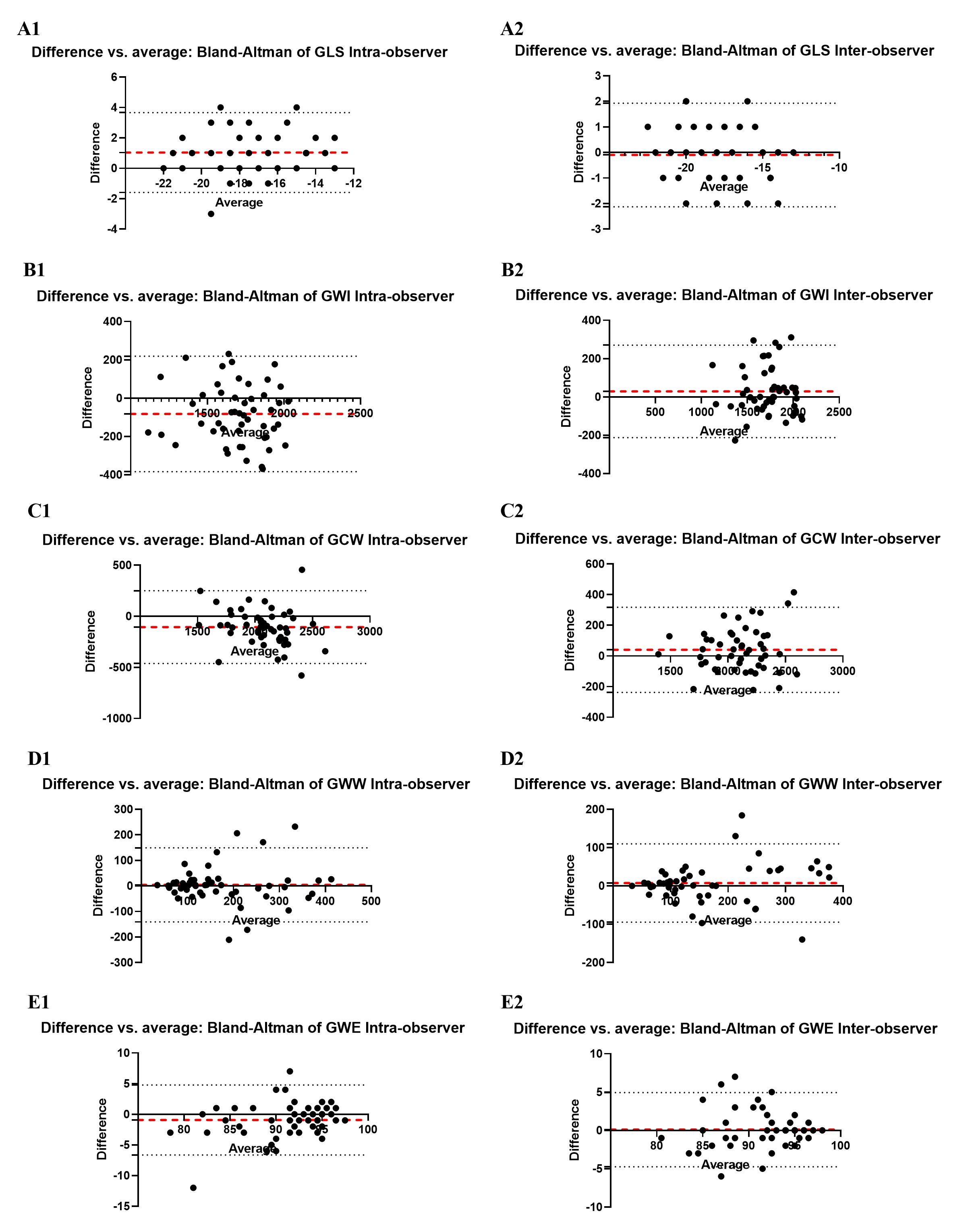

Supplement: Supplementary file 3 — Supporting information. [file CLC-47-e24172-s001.jpg]
